# CircRNAs in colorectal cancer: potential biomarkers and therapeutic targets

**DOI:** 10.1038/s41419-023-05881-2

**Published:** 2023-06-09

**Authors:** Yuying Zhang, Jingyan Luo, Weikang Yang, Wen-Chu Ye

**Affiliations:** 1 Central Laboratory, Shenzhen Longhua Maternity and Child Healthcare Hospital, Shenzhen, 518109 China; 2 Forevergen Biosciences Centre, Guangzhou International Biotech Island, Guangzhou, 510300 China; 3 Department of Prevention and Healthcare, Shenzhen Longhua Maternity and Child Healthcare Hospital, Shenzhen, 518109 China; 4 Shenzhen Research Institute, The Chinese University of Hong Kong, Shenzhen, China

**Keywords:** Colorectal cancer, Non-coding RNAs

## Abstract

Globally, colorectal cancer (CRC) is the third most prevalent cancer and the second leading cause of cancer-related deaths. Circular RNAs (circRNAs) are single-stranded RNA with covalently closed-loop structures and are highly stable, conserved, and abundantly expressed in various organs and tissues. Recent research found abnormal circRNA expression in CRC patients’ blood/serum, cells, CRC tissues, and exosomes. Furthermore, mounting data demonstrated that circRNAs are crucial to the development of CRC. CircRNAs have been shown to exert biological functions by acting as microRNA sponges, RNA-binding protein sponges, regulators of gene splicing and transcription, and protein/peptide translators. These characteristics make circRNAs potential markers for CRC diagnosis and prognosis, potential therapeutic targets, and circRNA-based therapies. However, further studies are still necessary to improve the understanding of the roles and biological mechanisms of circRNAs in the development of CRC. In this review, up-to-date research on the role of circRNAs in CRC was examined, focusing on their potential application in CRC diagnosis and targeted therapy, which would advance the knowledge of the functions of circRNAs in the development and progression of CRC.

## Facts

- CircRNAs are single-stranded RNA that may be used to treat or prevent colorectal cancer.
- CircRNAs may service as useful biomarkers for cancer diagnosis and therapy.
- CircRNAs are widely expressed in CRC patients’ blood/serum, cells, CRC tissues, and exosomes.
- Some circRNAs exert a cancer-promoting effect in CRC, while others are not.

## Open Questions

- What is the roles and biological mechanisms of circRNAs in the development of CRC?
- How do circRNAs contribute to the development and progression of CRC by controlling alternative splicing?
- Can circRNAs be utilized as biomarkers to predict chemotherapy resistance in CRC therapies?

## Introduction

Globally, colorectal cancer (CRC) is the third most prevalent cancer and the second leading cause of cancer-related deaths [1]. Recent estimates indicate that over 1.9 million new cases and 93,000 CRC-related deaths occurred in 2020, accounting for approximately 10% of all cancer cases and 9.4% of cancer-related deaths [2]. The early detection of CRC can help minimize morbidity and mortality; however, most CRCs are diagnosed at an advanced stage owing to the lack of distinct early symptoms, limiting the opportunity for effective early treatment. Therefore, it is imperative to identify new therapeutic targets and biomarkers for effective early detection, personalized treatment, and monitoring of CRC to improve prognosis.

Both genetic and epigenetic alterations can cause CRC. circRNAs, a novel type of non-coding RNAs, have been identified as tumor-initiating and tumor-progressing factors. Compared with linear RNAs, the closed structure of circRNAs makes them highly stable and conserved [3]. Recently, bioinformatics analysis of RNA-seq has facilitated the identification of several circRNAs in eukaryotes and shown that circRNAs have tissue-specific expression patterns [4]. Ongoing studies have revealed that dysregulation of circRNAs contributes to the development of various cancers, including CRC [5], and lung [6], liver [7], and bladder cancers [8]. Further investigations have identified several dysregulated circRNAs that play important roles in CRC progression [9]. Additionally, circRNAs are abundantly found in exosomes, human peripheral blood, and fluids, making them potential diagnostic biomarkers and therapeutic targets [10]. Therefore, circRNAs may serve as promising biomarkers for CRC.

This review highlights the current research progress on the biogenesis and characteristics of circRNAs and their mechanisms in CRC. Additionally, the therapeutic and diagnostic potentials of circRNAs in CRC were extensively discussed.

## Alternative modes of circular RNA splicing

**Fig. 1:**
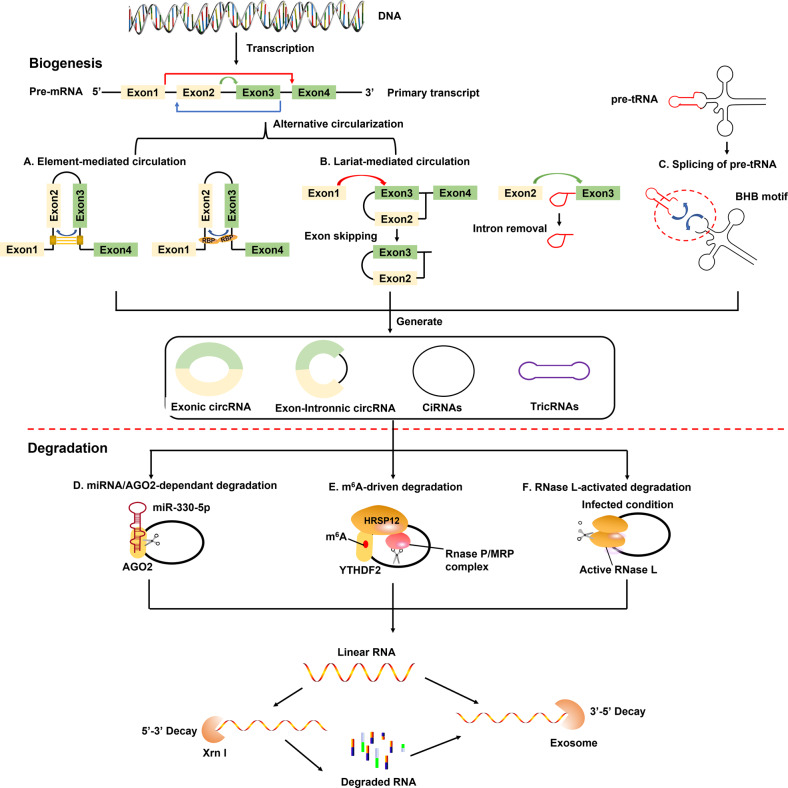
Biosynthesis and metabolism of circRNA. **A** Possibility of looped structures generation either by base-pairing among complementary sequences that flank the circularized exons or via RBPs. To generate EcRNAs and EIciRNAs, the intron sequences can be deleted or kept respectively in the loop structure. **B** The exon-skipping events generate certain EcRNAs, whereas a lariat is internally spliced to remove intronic sequences. **C** The intron-containing pre-tRNA is cleaved at the BHB motif into half of the exon and intron part. A mature tRNA is formed by joining the halves of the exons, and a tricRNA is produced by joining the termini of the introns. **D** miRNA directly binds at the AGO2-dependant cleavage site of targeted mRNA molecules in a complementary way. **E** RNase mitochondrial RNA processing (RNase MRP) promotes the cleavage of m^6^A-possessing circRNAs via the activities of YTHDF2 and HRSP12. **F** When infected by viruses, active RNase L degrades circRNA. AGO2 argonaute 2, BHB motif bulge-helix-bulge motif, ciRNA circular intronic RNA, EcRNAs exonic circRNAs, EIciRNAs exon-intron circRNAs, RBP RNA-binding protein, YTHDF2 YT521-B homology domain family 2, tricRNA tRNA intronic circular RNA.

RNA splicing is a fundamental and highly regulated process in the eukaryotic gene. The splicing of pre-messenger RNA (pre-mRNA) is catalyzed via spliceosome, a highly dynamical ribonucleoprotein (RNP) machinery that can remove introns and then join exons together to form mature mRNA [11] (Fig. 1). Alternative splicing involves the transcription of pre-mRNAs to generate different mature mRNAs depending on how they are spliced, thereby increasing protein diversity [12]. The normal pre-mRNA spliceosomal mechanism, which involves the back-splicing of intronic, exonic, or intergenic sequences, is necessary to synthesize circRNAs. By backsplicing pre-mRNAs, which requires the covalent interaction of an upstream 3′ splice site and a downstream 5′ splice site, circRNAs are selectively produced [13]. Through controlling alternative splicing pathways, circRNAs have been demonstrated to play crucial roles in carcinogenesis, according to mounting evidence [14–16]. For example, Wang et al. discovered circURI1, created by the back-splicing of exons 3 and 4 of URI1-14, an unusual prefoldin RPB5 interactor. CircURI1 may control alternative splicing to contribute to developing and spreading gastric cancer [13].

Numerous RNA-binding splicing factors (RBFOX1/2/3) contain an RNA-recognition motif that binds to this GCAUG element and affects the regulation of various alternate splicing events [14, 15]. Recent studies have shown that RBFOX proteins can either repress or activate alternate splicing, determining the binding location to pre-mRNA exon [16]. Suppression of a splicing regulator RNA binding protein fox-1 homolog 2 (RBFOX2) promoted preferential splicing of the mRNA isoform, such as KIF1B beta [17], TEAD1 [16], and TFRC [18]. Specifically, Zhang et al. found that circRAPGEF5 could interact with RBFOX2 and inhibit its binding to pre-mRNA, thereby causing exon exclusion of TFRC in endometrial cancer [18]. Moreover, although RBFOX2 is known to regulate some of these genes, the role of RBFOX2-mediated splicing events on signaling pathways in cancer remains largely unknown.

## circRNAs: biogenesis and characteristics

Based on their biogenesis mechanisms, circRNAs can be classified as EcircRNAs, EIciRNAs, ciRNAs, and mecciRNAs [19–21]. The biogenesis of circRNAs can be facilitated by pre-mRNAs containing a reverse complement of Alu repeat flanking the circularized exons [22]. RNA-seq and bioinformatic analysis has revealed the relationship between flanking introns and reverse complementary Alu repeats in mammalian circRNA biogenesis. CircRNA exons often have long flanking intronic sequences and repetitive Alu elements, both promoting circularisation by base pairing and reducing the distance between potential back-splicing sites [23, 24]. Moreover, loss of flanking Alu repeats inhibited the circularization of circRNA in vitro, including CircERBB2 [25]. ALU repeats present an underestimated risk, while enzymes such as adenosine deaminases acting on RNA (ADARs) and DExH-Box Helicase 9 (DHX9) are critical in destabilizing intron pairing during the biogenesis of circRNAs [26, 27]. A study by Shen et al. characterized ADARs as potent regulators of circular transcriptomes by identifying over a thousand circRNAs in a bidirectional manner [28]. The biogenesis of circRNAs and ADARs has been found to be negatively correlated in recent studies. For example, the knockdown of ADAR1 increased the intracellular circRNA expression in the mammalian brain [29]. Furthermore, ADARs-regulated circRNAs are ubiquitously expressed in numerous cancer types, suggesting high functional relevance to cancer [28]. Additionally, DHX9 deletion increased a subset of circRNA-producing genes and amounts of circular RNA, repeat Alu elements, and transcriptional rewiring of susceptible loci [26]. In addition to the above-mentioned examples, RNA binding proteins (RBPs), including muscleblind (MBL/MBNL1) and quaking (QKI), have been shown to promote the biogenesis of circRNAs [30, 31]. Ashwal-Fluss et al. showed that ectopic expression of MBL/MBNL1 increased the expression of circMbl by binding to flanking introns; however, downregulation of MBL/MBNL1 significantly decreased CircMbl expression [24]. Thus, MBL/MBNL1 was involved in circRNA biogenesis. Another important RBP, QKI, can also positively regulate circRNA biogenesis. For instance, knockdown of QKI inhibited circRNAs expression during circRNA biogenesis, however, overexpression of QKI lead to the circRNA biogenesis in human immortalized mammary epithelial cells [30]. Thus, the biosynthesis of circRNAs is influenced by QKI.

## CircRNA mechanisms of action

**Fig. 2:**
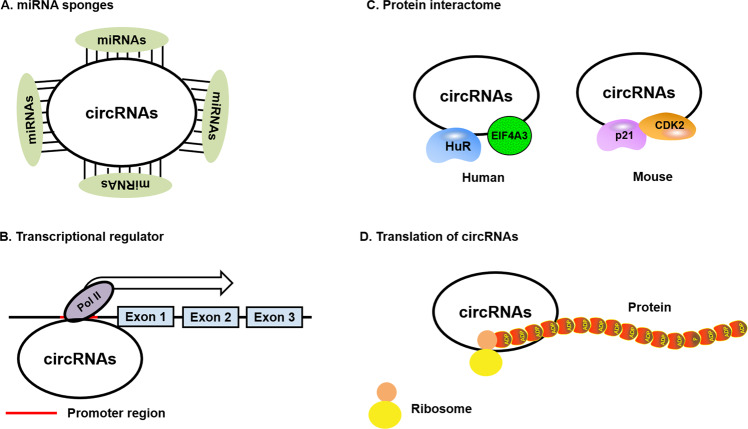
Mechanisms of circRNA functions. **A** CircRNAs can act as sponges or decoys for miRNAs. MiRNA binding to circRNAs may release target mRNAs from miRNA-dependent degradation, resulting in more effective translation. **B** circRNAs containing RBP motifs (such as HuR, EIF4A3, P21, and CDK2) may act either as sponges or decoys for the aforementioned proteins while regulating their functions. **C** circRNAs may interact with the RNA polymerase II (Pol II) complex containing the U1 snRNP in the promoter region of targeted genes, and significantly enhance its function. **D** circRNAs contain ribosome entry sites that may be translated to produce unique peptides under specific conditions.

Recent studies have demonstrated that circRNAs can interact with miRNAs, and RBPs serve as protein baits or antagonists to exert the functions of circRNAs [32]. As regulators of gene expression, circRNAs are involved in several biological processes, including miRNA sponges [33], transcription and translation, RBPs, and translation of peptides and proteins [34] (Fig. 2).

## miRNA sponges

Little non-coding RNAs called miRNAs play an important role in physiological and pathological processes. They typically have a base length of 21–25 nucleotides. CircRNAs may behave as miRNA sponges, limiting miRNA action in the transcriptional and post-transcriptional control of gene expression (for example, mRNA stability) [35]. Cancer cells’ proliferation, migration, and angiogenesis have all been linked to this sponge process. For instance, by functioning as a sponge for miR-328-5p and reversing its repression of E2F1 [36], circSHKBP1 facilitated the advancement of CRC. By activating the miR-142-3p/miR-506-3p-TGF-1 axis, CRC-derived exosomal circPACRGL promoted cell proliferation, migration, invasion, and neutrophil differentiation [8]. This method has also been described in other fields. For instance, circHIPK3 promoted the development of retinal vascular dysfunction in diabetes mellitus by sponging miR-30a-3p [37] and modulated the autophagy in STK11 mutant lung cancer by sponging miR-124-3p [38]. In addition, circHIPK3 also suppressed CRC growth and metastasis by sponging miR-7 [35]. However, some circRNAs have been shown to function in multiple roles by sponging different miRNAs. CircSLC8a1 exacerbated myocardial injury by sponging miR-214-5p [36] and inhibited the progression of non-small cell lung cancer (NSCLC) by sponging miR-106b-5p [39].

## Transcription and translation

CircRNAs can regulate gene transcription in both a direct and indirect manner. Moreover, certain circRNAs have been reported to modulate gene transcription by interacting with the RNA polymerase II complex and translating associated proteins. For instance, circEIF3J and circPAIP2 promoted PAIP2 and EIF3J transcription by interacting with U1 small nuclear ribonucleoprotein (snRNP) and RNA polymerase II [40]. However, circRNAs are mainly cis-regulators of transcription in various physiological and pathological processes. For instance, circRHOT1 inhibited the progression of hepatocellular Carcinoma (HCC) via recruiting TIP60 to the NR2F6 promoter and subsequently initiating the transcription of NR2F6 [41]. circMEMO1 can regulate TCF21 promoter methylation and its gene expression to promote the progression of HCC [42]. In another study, circAmotl1 promoted skin wound repair by increasing STAT3 expression and nuclear translocation [43]. It may be possible to provide clinical insight into skin wound healing by the ectopic application of circ-Amotl1.

Recently, Pamudurti et al. demonstrated that translating ribosomes is associated with a set of circRNAs through ribosome footprinting from fly heads [44]. Mass spectrometry also detected a protein encoded by circRNA generated from the muscleblind locus [44]. Additionally, exosomal circLPAR1 directly bound to eIF3h and specifically suppressed the Interaction between METTL3 and eIF3h, which caused BRD4 translation to decrease [45]. CircVAMP3 interacted with CAPRIN1 and G3BP1 to trigger phase separation of CAPRIN1 and promoted stress granule formation [46]. CircVAMP3 can reduce the protein level of Myc proto‐oncogene protein by inhibiting c‐Myc translation. Hence, circVAMP3 suppressed tumor growth in HCC by inhibiting the translation of c‐Myc.

## RBPs

Besides their miRNA sponge and transcription and translation function, circRNAs with RBP binding sites may function as protein sponges or decoys in regulating gene expression [47]. CircRNAs can directly interact with one or different proteins and act as “scaffolding” protein complexes to form an RNA-protein complex. For example, circRHOBTB3 suppressed lung metastasis by binding to the HuR protein and promoting β-Trcp1-mediated ubiquitination of HuR, improving the stability of polypyrimidine tract-binding protein 1 (PTBP1) [48]. In cancer cells, Chen et al. found circAGO2 promoted cancer progression by interacting with HuR protein and inhibiting the functions of AGO2-miRNA complexes [49]. In addition, circRNA can also affect their biological function by sequestering proteins [50]. For example, circPABPN1 reduced ATG16L1 production by inhibiting HuR binding to Atg16l1 mRNA [51]. However, scaffolding enhanced direct protein interactions in contrast to their sequestering function. For example, circFOXO3 functioned as a scaffolding molecule that enhanced the Interaction between CDK2 and p21 [52]. CircACC1 can directly bind to the β and γ subunits of AMPK, enhancing its stability and enzymatic activity [53].

## Peptides and proteins

Based on bioinformatics platforms and computational analysis, some circRNAs have open reading frames (ORFs) and can encode proteins [44]. Some other circRNAs also encode proteins or peptides with tumor-suppressive or oncogenic properties. Presently, hundreds of peptides encoded by circRNAs have been detected by liquid chromatography coupled with mass spectrometry (LC-MS), indicating that circRNAs can translate proteins. CircPLCE1 encoded a novel circPLCE1-411 protein that inhibited tumor proliferation and metastasis in CRC cells [54]. Exosomal CircATG4B encoded a novel protein and induced oxaliplatin resistance in CRC by promoting autophagy [55]. In addition, circular RNAs can be classified as IRES-dependent or IRES-independent translational machinery. For example, circSHPRH can encode an SHPRH-146aa protein in an IRES-dependent manner [56]. It was found that SHPRH-146aa is a tumor suppressor protein that prevents SHPRH full-length protein from degradation [56], suggesting that aberrant translation of circSHPRH affects tumor malignancy. Lastly, some circRNAs are capable of encoding peptides without requiring IRES. Recent studies have discovered that consensus N6-methyladenine (m^6^A) modification motifs are enriched in circRNAs, and one m^6^A site can initiate translation [57]. For example, YTHDF3, an m^6^A reader protein, modulates circ-ZNF609 to translate two proteins using two alternative START codons [58]. Overall, the protein-coding capacity of circRNA is of great significance to human disease diagnosis and treatment.

## Dysregulation of circRNA in CRC

**Fig. 3:**
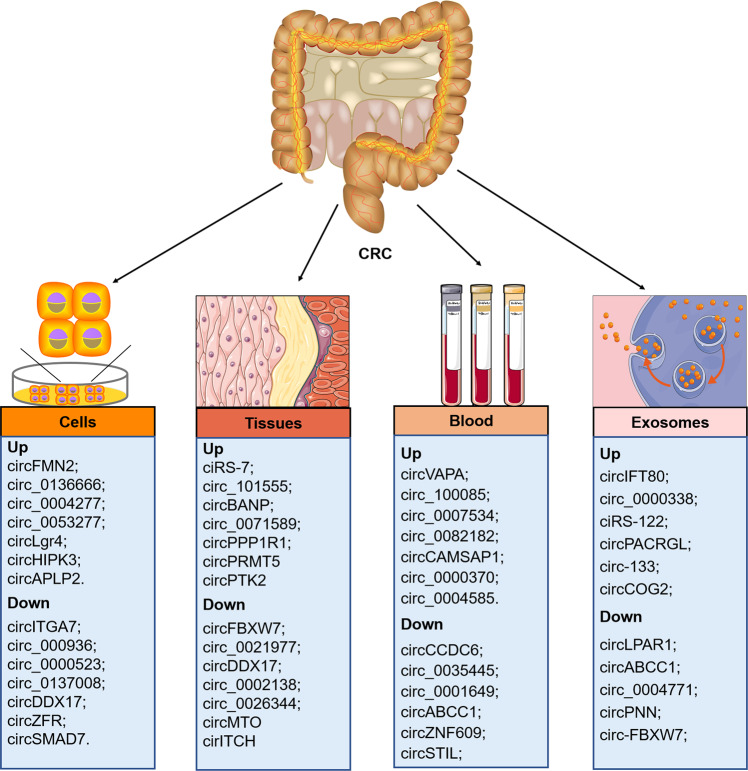
Aberrant expressions of circRNAs in CRC patients. circRNAs are aberrantly expressed in blood/serum, cells, CRC tissues, and exosomes from CRC patients. circRNA dysregulations are highlighted in the figure; ‘Up’ indicates upregulated, ‘Down’ indicates downregulated.

Recently, the roles of circRNAs in tumorigenesis and other diseases have received considerable research attention. Accumulating evidence indicates that abnormalities in circRNAs are associated with colorectal malignancies (Table 1; Fig. 3). For instance, circHERC4 was highly elevated in CRC tissues and positively associated with lymph node metastasis and advanced tumor [59]. In contrast, circPLCE1 was downregulated in CRC tissues and was linked to poor survival and advanced clinical stages [54]. Interestingly, the expression levels of circRNAs vary in different cancer types, indicating their distinct biological roles. For example, circHIPK3 was significantly upregulated in gastric cancer, HCC, breast cancer, CRC, and lung cancer tissues and cell lines [35, 60–62], but downregulated in bladder cancer [63]. Although several studies have reported abnormal expression of circRNAs in various cancers [6]; the possible causes of circRNA dysregulation remain largely unknown and require further investigation.

**Table 1:** Summary of dysregulated circRNAs in colorectal cancer.

Mechanism	CircRNAs	Annotated number	Expression	Samples	Targets/ Regulators	Function	Ref
miRNA sponge	CircPACRGL	has_circ_0069313	Up	CRC cells	miR-142-3p/506-3p	Promotes proliferation, migration, and invasion.	[8]
	CircCCDC66		Up	Tissues	miR-33b/93	Promotes proliferation, and metastasis.	[137]
		hsa_circ_0001313			miR-370	Promotes proliferation, migration, and invasion	[138]
	Circ_0000392	hsa_circ_0000392	Up	Tissues	miR-193a-5p	Promotes proliferation, and invasion.	[74]
	ciRS-122	hsa_circ_0005963	Up	CRC cells	miR-122	Promotes chemoresistance.	[98]
	Circ_101277	hsa_circ_101277	Up	Tissues	miR-370	Promotes proliferation and cisplatin resistance.	[108]
	CircATAD1	hsa_circRNA_100641	Up	Tissues	miR-618	Promotes proliferation.	[139]
	CircMET	hsa_circ_0082002	Up	Tissues; CRC cells	miR-410-3p	Promotes proliferation and growth.	[140]
	Circ_0000467	hsa_circ_0000467	Up	Tissues; CRC cells	miR-330-5p	Promotes proliferation, migration, and invasion.	[141]
	CircN4BP2l2	hsa_circ_0000471	Up	Tissues; CRC cells	miR-340-5p	Promotes migration, invasion, proliferation	[103]
	Circ_0068464	hsa_circ_0068464	Up	Tissues; CRC cells	miR-383	Promotes tumor growth and lung metastasis	[86]
	Circ_0000231	hsa_circ_0000231	Up	Tissues	miR-375	Promotes proliferation, and tumorigenesis.	[142]
	CircRAD23b	hsa_circ_0087862	Up	Tissues; CRC cells	miR-1205	Promotes proliferation, and metastasis	[143]
	Circ_0011385	hsa_circ_0011385	Up	Tissues	miR-330-3p	Promotes proliferation, migration, and invasion.	[144]
	Circ_0006732	hsa_circ_0006732	Up	Tissues; CRC cells	miR-127-5p	Promotes proliferation, migration, invasion, and EMT	[102]
	Circ_0007919	hsa_circ_0007919	Up	Tissues; CRC cells	miR-942-5p	Promotes growth and migration	[145]
	Circ_0014130	hsa_circ_0014130	Up	Tissues; cells	miR-197-3p	Promotes proliferation.	[146]
	Circ_0101802	hsa_circ_0101802	Up	Tissues; cells	miR-665	Promotes proliferation, migration, and tube formation.	[147]
	CircALG1	hsa_circ_0037777	Up	Tissues; blood	miR-342-5p	Promotes metastasis, migration, and invasion	[81]
	CircSMAD4a	hsa_circ_0004846	Up	Tissues	miR-545-3p	Promotes proliferation.	[148]
	Circ_0001955	hsa_circ_0001955	Up	Tissues	miR-583	Promotes tumor growth.	[149]
	CircFAT1	hsa_circ_0001461	Up	Tissues	miR-619-5p	Promotes migration, invasion, and angiogenesis.	[150]
	CircLHFPL2	hsa_circ_7431	Up	Tissues	miR-556-5p/1322	Promotes CRC progression.	[109]
	CircTMEM59	hsa_circ_0012634	Down	Tissues; cells	miR-668-3p	Inhibits growth and metastasis.	[151]
	Circ-PLXNB1	hsa_circ_0065378	Down	Tissues	miR-4701-5p	Inhibits proliferation, cell invasion, migration, and EMT.	[104]
Protein decoy	CircACC1	hsa_circ_001391	Up	Tissues	AMPK	Promotes proliferation.	[53]
	CircREEP3	hsa_circ_400564	Up	Tissues	FKBP10	Promotes tumorigenesis and metastasis.	[152]
	CircLPAR1	hsa_circ_0087960	Down	Tissues	BRD4	Inhibits tumor growth.	[45]
	CircPTEN1	hsa_circ_0002232	Down	Tissues	TGF-β/Smad signaling	Inhibits metastasis, and invasion.	[85]
	Circ-LECRC	has_circ_0004140	Down	Tissues	YAP signaling	Inhibits proliferation, migration, and invasion and promotes apoptosis.	[153]
	CircRHOBTB3	hsa_circ_00074444	Down	Tissues; cells	HuR	Suppresses tumor metastasis	[48]
	Circ_0014717	hsa_circ_0014717	Down	Tissues; cells	p16	Inhibits cell proliferation and colony formation	[154]
	CircRIP2	has_circ_0005777	Up	Tissues; cells	CBFB	Aggravates CRC deterioration	[155]
Coding peptides	CircPLCE1	hsa_circ_0019223	Down	Tissues; cells	NF-κB, HSP90α/RPS3 complex	Inhibits CRC proliferation and metastasis.	[54]
	Circ_0000725; circ_0008826	hsa_circ_0000725; hsa_circ_0008826	Up	Tissues	306-a.a. and 475-a.a. peptide	Promotes proliferation, and invasion.	[156]
	Circ_0006401	hsa_circ_0006401	Up	Tissues	circ_0006401 peptide	Promotes CRC proliferation and migration	[157]
	CircPPP1R12a	hsa_circ_0000423	Up	Tissues	circPPP1R12A-73aa	Promotes cell proliferation, migration, and invasion.	[100]

*AMPK* AMP-activated protein kinase, *BRD4* bromodomain containing 4, *CBFB* core-binding factor subunit beta, *CRC* colorectal cancer.

*EMT* epithelial-mesenchymal transition, *FKBP10* FKBP prolyl isomerase 10, *hnRNP A1* heterogeneous nuclear ribonucleoprotein A1, *HSP90a* heat shock protein 90 alpha. *HuR* human antigen R, *NF-κB* nuclear factor kappa B, *RPS3* ribosomal protein S3, TGF-β transforming growth factor-beta.

The dysregulated circRNA expression in CRC could be due to abnormal expression of host genes, such as chromosomal amplification, depletion, or translocation. CircPRKCI is a proto-oncogenic circRNA located in the 3q26.2 amplicon in several cancers, including lung cancer, glioma, and esophageal cancer [64]. Therefore, circPRKCI upregulation in cancers may be due to PRKCI amplification. Fusion-circRNAs (F-circRNAs) are products of cancer-associated chromosomal translocations in host genes [65]. For example, two novel circRNAs (F-circSR1 and F-circSR2) generated from oncogenic SLC34A2-ROS1 fusion gene, promoted cell migration in non-small cell lung cancer [66]. Additionally, F-circEA generated from oncogenic EML4-ALK fusion facilitated cell migration and invasion in lung cancer [67]. The fusion genes produce functional proteins that contribute to oncogenesis.

Several studies have demonstrated the elimination of circRNAs by RNase L following the release of extracellular vesicles, viral infection, or poly I: C stimulation. Degradation of circRNAs by RNase L activation can increase protein kinase R (PKR) phosphorylation. Apart from degradation, circRNAs can also be eliminated from cells via exocytosis. Additionally, circRNAs in exosomes may be involved in the communication mechanism, indicating the need for further studies on the degradation and extracellular transportation of circRNAs. Moreover, circRNAs are abundant in the cytoplasm and can be transported to exosomes from the cytoplasm.

Recently, some studies reported that RNA modification at the m^6^A site was associated with circRNA degradation, translation, and expression in cancer cells [68]. The most prevalent internal alteration associated with eukaryotic ncRNAs is m^6^A, which influences RNA stability, splicing, export, translation, and degradation, all of which affect biological activities. MALAT1 is highly methylated at m^6^A, and its two residues can block local RNA structure formation and facilitate the recognition and binding of heterogeneous nuclear ribonucleoprotein C (HNRNPC) through an “m^6^A switch” mechanism [68]. m^6^A alteration accelerated circNSUN2 transport to the cytoplasm and increased the stability of HMGA2 mRNA to induce CRC metastasis by creating a circNSUN2/IGF2BP2/HMGA2 complex in the cytoplasm [69]. Recently, m^6^A-modified circRNAs have been identified using cell-type-specific methylation patterns. m^6^A recruits YTHDF3 and eIF4G2 to regulate protein synthesis from circRNAs. ALKBH5 (m^6^A eraser) and METTL3 (m^6^A writer) can affect circRNA biosynthesis by altering the m^6^A levels. For instance, the m^6^A levels of circRNA-SORE were elevated in sorafenib-resistant cells and downregulated when m^6^A modification was suppressed [70]. Recent findings showed that circCUX1 expression was stabilized by METTL3-mediated m^6^A modification [71]. Thus, the loss of m^6^A sites or removal of m^6^A from circRNAs can decrease their methylation levels, resulting in the dysregulation of circRNAs. Furthermore, the deregulation of critical components during the degradation of circRNAs might result in abnormal circRNA expression.

Super enhancers (SEs) comprises of large putative enhancer clusters that are enriched to bind key master transcription factors. These enhancer clusters play key roles in driving tumorigenesis and act as causal mechanisms for regeneration by regulating circRNA expression. SEs are frequently dysregulated in cancer and are central to the maintenance of cancer cell identity. SEs are important for controlling tumor metastasis, proliferation, and chemoresistance. SEs are abnormally activated in various tumors, regulate key target genes in cancer, and promote tumorigenesis and development. For example, EphA2-SE at core active regions contains an E1-enhancing component that induces cell proliferation and metastasis via the involvement of TCF7L2 and FOSL2 to upregulate EphA2 expression [72]. RNA-seq combined with in vitro functional experiments revealed that EphA2-SE deletion mediated the suppression of cell growth and metastasis in HCT-116, HeLa, and MCF-7 cells, whereas EphA2 overexpression in EphA2-SE^−/−^ clones reversed EphA2-SE knockdown-induced effects on cell proliferation and metastasis [72]. Recent studies have shown that circRNAs are potential SEs that modulate gene expression and are involved in the pathogenesis of several diseases. Thus, SEs are likely to control tumor metastasis and chemoresistance by controlling circRNA expression.

## CircRNAs participate in the progression of CRC

Tumor invasion and metastasis are multistep, complex dynamic processes involving growth, invasion, metastasis, and intravasation, and are responsible for most CRC-associated mortalities. Transcriptome analysis identified 80 differentially expressed circRNAs, including 33 upregulated and 47 downregulated circRNAs, between CRC and para-cancerous tissues. Circ3823 and circRNA_0000392 were significantly upregulated in CRC tissues and cell lines, indicating that higher circ3823 and circRNA_0000392 expression levels could predict poor prognosis in CRC patients [73, 74]. Several studies have shown that circRNAs can regulate CRC metastasis primarily by influencing key factors that regulate several pathways closely associated with CRC metastasis. The mechanism of circRNAs in CRC metastasis is diverse and includes acting as miRNA sponges, interacting with RBPs, regulating gene splicing or transcription, translating proteins, and regulating epigenetics.

CircRNAs are stable in tissues and cells owing to their closed-loop structure with no 5′ or 3′ ends, thereby preventing ribonuclease degradation. Additionally, circular sequences include several miRNA response elements that facilitate the binding of circRNAs and miRNAs. CircRNAs might therefore function as natural miRNA sponges to modulate target gene expression. For example, circ3823 acted as a sponge for miR-30c-5p and regulated its target TCF7 expression, which increased the expression of MYC and CCND1 and promoted CRC progression [73]. Additionally, circRNA_0000392 promoted the proliferation and invasion of CRC cells through the miR-193a-5p/PIK3R3/Akt axis [74], indicating the potential of circRNA_0000392 as a prospective therapeutic target for CRC therapy as well as a prediction marker. However, most circRNAs are less abundant than miRNAs and may fail to meet the stoichiometric requirement for a sponge effect.

In addition to acting as miRNA sponges, certain circRNAs with RBP binding sites may act as protein sponges or decoys. Studies have shown that circRNAs can bind to RBPs, such as Quaking (QKI), HuR (ELAVL1), eukaryotic translation initiation factor 4A3 (EIF4A3), and AlkB homolog H5 (ALKBH5), to play important roles in tumor progression. circRNAs can interact with regulatory RBPs to influence the destiny of their target mRNAs. For example, circRHOBTB3 acted as a HuR sponge and facilitated HuR-mediated PTBP1mRNA stability [48], indicating that circRHOBTB3 exerted a suppressive effect on CRC. An increase in AMPK activation in CRC tissues is related to elevated expression of circACC1, and circACC1 has been demonstrated to stabilize and enhance AMPK holoenzyme activity by forming a complex with regulatory β and γ subunits [53]. RNA pull-down and RNA immunoprecipitation (RIP) assays showed that hsa_circ_0068631 can bind to EIF4A3 and recruit EIF4A3 to increase c-Myc mRNA stability in breast cancer [75]. Additionally, *cIARS* (hsa_circ_0008367) physically interacted with ALKBH5 and markedly promoted sorafenib-induced ferroptosis in HCC via inhibition of ALKBH5-mediated autophagy [76]. Recent research suggests that distinct RBPs may play varied or opposing functions in the back-splicing process. For instance, circRNA synthesis can be enhanced by the RBPs highlighted above; in contrast, circRNA synthesis can be inhibited by the RNA-editing enzyme ADAR1. ADAR1 significantly and specifically inhibited the biogenesis of circHIPK3, which altered the precursor of circHIPK3 secondary structure [77].

## CircRNAs as potential biomarkers for CRC diagnosis and prognosis

**Fig. 4:**
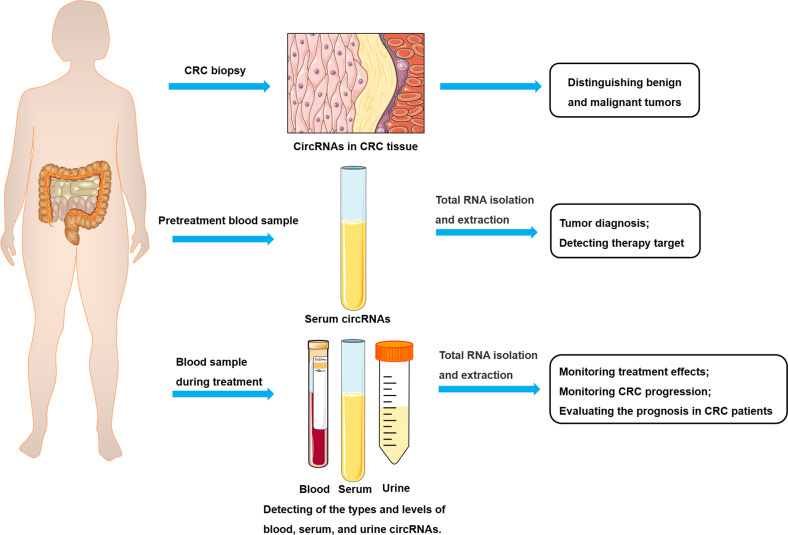
circRNAs as potential biomarkers for the diagnosis and treatment of CRC. circRNAs can act as an indicator of the differentiation of benign and malignant tumors. Serum circRNAs levels can act as potential biomarkers in CRC diagnosis, providing information for the selection of appropriate therapeutic strategies by clinicians. The circRNA levels of blood, serum, and urine samples could also serve as important clinical markers for CRC monitoring, treatment, and prediction of patient outcomes.

CRC screening and early detection are crucial in enhancing treatment effectiveness and reducing CRC-related mortalities. CRC can be undetected over a long period, and only a few cases are diagnosed after presenting classic symptoms, such as weight loss, change in bowel habits, and perirectal bleeding. However, although stool occult blood tests, electronic colonoscopy, and digital rectal examination have improved the detection of CRC, effective biomarkers for CRC are necessary for early detection. Identifying either blood/serum or urine-based epigenetic biomarkers could be a promising diagnostic tool, as it would be noninvasive and inexpensive (Fig. 4).

The sensitivity and specificity of circRNAs provide a valuable biomarker for CRC diagnosis for several reasons. First, due to their lack of 5′ or 3′ prime ends, circRNAs are highly resistant to exonuclease degradation and are extremely stable; thus, they are highly specific for tissues and diseases [78]. Secondly, circRNAs are found in cancer cells, solid tumors, peripheral blood, exosomes, and body fluids such as serum, plasma, and saliva [79]. For example, circ1662 and circPACRGL were significantly higher in patients with CRC, implying their specificity to cancer [8, 80]. Due to their resistance to degradation and presence in body fluids, circRNAs are the perfect candidate for noninvasive liquid biopsy, and therefore they have a high diagnostic potential. Recently, circ-KLDHC10 was successfully detected in serum samples, which can be used to distinguish patients with and without CRC [78]. A high level of CircALG1 expression was observed in CRC patients’ peripheral blood and tumor tissues and correlated with CRC metastasis [81], suggesting it may be an important biomarker for cancer. The importance of circRNAs as biomarkers for CRC diagnosis and prognosis is emphasized in this research because they regulate cancer signaling pathways. Numerous studies have demonstrated the critical roles that circRNAs play in cancer signaling pathways, including the PI3K/Akt, JAK/STAT, GEF-H1/RhoA, Wnt/-Catenin, and TGF-/Smad pathways, by upregulating oncogene expression, downregulating tumor suppressor genes, and/or downregulating downstream proteins [82–86]. Thus, circRNAs may be a valuable biomarker for CRC diagnosis.

CircRNAs possess potential applications as clinical biomarkers for liquid biopsies considering their resistance to RNase R digestion, presence as covalently closed continuous loops, and wide existence in eukaryotes. Particularly, circRNAs are promising biomarkers for the clinical diagnosis and prognosis of cancer because they can easily be detected using qualitative real-time PCR (qRT-PCR), and are highly stable and abundant in bodily fluids, such as serum/blood, saliva, and urine (Fig. 4). Current findings have shown that circRNAs exhibit aberrant expression, increased disease specificity, and clinical implications, making them potential candidates for CRC diagnosis. For example, circ3823 upregulation in CRC tissues was correlated with increased cell proliferation, metastasis, and angiogenesis and was an independent predictor of poor prognosis in patients with CRC [73]. Human circRNA microarray analysis indicated an increase in the expression of 30 circRNAs between CRC and normal tissues, which may be used as prognostic biomarkers for overall survival [87]. Additionally, five circRNAs (circ_0003906, circCDC66, circITGA7, circ_0000567, and circ_0001649) have been identified in CRC tissues and clinically validated using qRT-PCR and RNA-seq [88], and area under the curve (AUC) values were 0.818, 0.884, 0.879, 0.865, and 0.857, respectively, indicating their potential as diagnostic biomarkers. Notably, circ_0001178 had an AUC value of 0.945 [88], indicating a highly effective value for accurate diagnosis. Additionally, circPTK2 overexpression in CRC tissue and blood serum was positively linked to metastasis, clinical stage, and chemoresistance [87]. Moreover, circ5615 upregulation in CRC tissues was highly correlated with T stage and poor prognosis in CRC patients [89]. However, circPTEN1 is downregulated in the peritumoral and tumor tissues of patients with CRC [85]. Decreased expression of circRNA has been reported to facilitate metastasis and promote cell invasion in PDX models and is an independent predictor of poor survival in patients [85]. These findings suggest that circRNAs possess promising applications as diagnostic biomarkers for CRC.

Furthermore, certain circRNAs possess potential applications as prognostic biomarkers for CRC. circHIPK3 significantly promoted CRC cell proliferation, migration, and invasion, induced apoptosis in vitro, and facilitated CRC growth and metastasis in vivo [62]. Additionally, circHIPK3 was highly upregulated in CRC tissues and cell lines, and positively correlated with metastasis and advanced clinical stages, indicating the potential of circHIPK3 as a predictive biomarker of CRC. Similarly, Geng et al. observed that circ_0009361 downregulation promoted proliferation, migration, invasion, and epithelial-mesenchymal transition (EMT) in CRC cells [90]. In contrast, circ_0009361 overexpression significantly inhibited CRC growth and metastasis, indicating that circ_0009361 could act as a prognostic biomarker for CRC. Additionally, circSPARC expression was upregulated in CRC cells and positively correlated with advanced tumor node metastasis (TNM) stage, lymph node metastases, and poor survival in patients [83]. Furthermore, correlation analysis indicated that circSPARC expression was associated with tumor size, invasion, lymphatic metastasis, distant metastasis, and clinical stage [83]. Kaplan-Meier analysis showed that high circSPARC levels were associated with a decrease in overall survival [83], indicating its potential as a predictive biomarker for CRC.

These findings confirm that certain circRNAs possess promising potential as diagnostic biomarkers for CRC. However, although these circRNAs are differentially expressed in tissues, they cannot be detected in the blood/plasma or serum. Therefore, detecting circRNAs circulating in liquid biopsies, such as blood/plasma and/or serum, could facilitate the development of valid test procedures to distinguish between CRC patients and healthy individuals.

However, several limitations are associated with the clinical application of circRNAs for cancer as biomarkers. Circular RNAs are difficult to detect since their sequences are nearly identical to linear RNAs. Circular RNAs must be distinguished from linear RNA species using appropriate methods, and these methods need to be sensitive enough to detect the closed-loop structure of circRNAs efficiently. For example, circular RNAs can be detected with qRT-PCR, but when primers are designed using a linear genome as a template, circRNAs cannot be distinguished from linear RNAs. Microarray technology is an effective and relatively sensitive technique for quantifying circRNA expression, but it can only detect known circRNAs and cannot detect unknown circRNAs [91]. Apart from the qRT-PCR and Microarray technology, high-throughput sequencing techniques have become increasingly popular, such as second-generation high-throughput sequencing (NGS) and third-generation high-throughput sequencing (HTS). Thousands of circRNAs in human cells have recently been identified by applying high-throughput RNA sequencing technology and bioinformatics methods [92].

Although several circRNAs can be potential tumor biomarkers, studies investigating circRNAs as CRC biomarkers are limited. Due to their lack of sensitivity or specificity, most circRNA biomarkers are unlikely to be suitable for clinical application. Importantly, numerous clinical studies should be conducted on circRNAs as biomarkers will require standardized techniques and bioinformatics methods for their detection. For example, sophisticated large-scale prospective studies involving collecting serial samples and establishing time points and intervals are necessary before circRNAs can be used as biomarkers. Additionally, circRNA standardization is a crucial factor in liquid biopsies. Repeated experiments are necessary to determine the optimal time and cutoff value for circRNAs to be consistent with patient demands. Regardless of the challenges, these findings suggest that circRNAs possess promising applications as diagnostic biomarkers for CRC.

## CircRNAs as potential therapeutic targets

More than 70 upregulated circRNAs are actively involved in CRC tumorigenesis and progression, and silencing them exerts opposite effects in vitro and in vivo [93]. Thus, these oncogenic circRNAs may serve as potential therapeutic targets, and target oncogenic circRNAs’ unique back-splice junctions for degradation by siRNAs may have anti-tumor properties. Numerous animal studies have revealed that siRNAs or short hairpin RNAs (shRNAs) specifically targeting oncogenic circRNAs have been shown to effectively inhibit the growth, proliferation, and metastasis of CRC [81, 94, 95]. For example, treatment with an shRNA targeting circMETTL3 inhibited tumor growth and metastasis in nude mice xenograft models [96], suggesting that the oncogenic circMETTL3 may serve as a potential therapeutic target. A PDX model for tumor metastasis was used by Chen et al. to confirm that the knockdown of circNSUN2 significantly reduced tumor metastasis in either liver or lung metastasis models [69].

Similarly, targeting circLONP2 by antisense oligonucleotide (ASO) significantly reduced the penetrance of CRC metastasis to foreign organs in vivo, including a reduction in both nodule size and number [97]. Interestingly, Wang et al. confirmed that an exosome-delivered siRNA targeting hsa_circ_0005963 sensitized CRC-resistant mice [98], implying a novel approach for reversing oxaliplatin resistance in CRC. Furthermore, certain drugs and compounds may exert anticancer activity through circRNA-associated pathways. For instance, lidocaine treatment inhibited the proliferation, and metastasis and induced cell apoptosis via regulating the circITFG2/miR-1204/SOCS2 axis, providing a novel treatment in improving CRC therapy [99]. Peptide 17 is a YAP-specific inhibitor that significantly inhibited the proliferation and metastasis-promoting effect of circPPP1R12A-73aa on colon cancer cells [100].

It is well known that numerous downregulated circRNAs negatively regulate CRC growth and metastasis. Due to their high stability and long half-life, tumor suppressor circRNAs may have significant anti-tumor effects when expressed in CRC cells or tissues. Zheng et al. observed that circLPAR1 was downregulated in CRC tissues and circLPAR1 overexpression treatment reduced tumor weight and size, implying that it portends poor prognosis [45]. Moreover, exogenous circRNAs may be delivered by specific vectors containing gene expression cassettes designed to express circRNAs or by transfection of purified in vitro-generated circRNAs. Recent studies have confirmed the synthesis and cloning of circRNA sequences into special vectors (such as lentiviruses vectors [LV] and recombinant adeno-associated viral [AAV] vectors) to produce LV or AAV to transfect CRC cell lines or animal model and constitutively overexpress the desired circRNAs [101]; the exogenous circRNA then acted as a tumor suppressor by sponging multiple miRNAs [102–104]. Engineered circRNAs could serve as sponges for specific oncogenic miRNAs in CRC cells or tissues, representing an efficient and innovative treatment approach for the disease in the future.

Some circRNAs such as circRS-122, circ_001680, circ_0002813, circ_101277, circ_0000236, and circ-ZEB1 are associated with chemotherapy resistance (e.g., fluorouracil (5-Fu), oxaliplatin, cisplatin, and irinotecan) in CRC [55, 98, 105–108]. For this reason, detecting the expression of these circRNAs may be useful for predicting the sensitivity of patients with CRC to chemoradiotherapy in the clinic. In addition, some circRNAs, such as circIFNGR2 and circLHFPL2, are related to drug resistance (e.g., cetuximab and MEK inhibitor) in CRC [95, 109]. Circular RNAs may be useful for predicting drug resistance in patients with CRC. Moreover, therapy targeting these circRNAs may also improve chemoradiotherapy and drug resistance in patients with CRC. For the treatment of CRC, antisense oligonucleotides (ASOs) were also developed that target circularization and secretion elements of circRNAs, including circRHOBTB3 [22]. Immunotherapy for CRC patients might be more effective with interventions targeting circular RNA CDR1-AS by PD-1/PD-L1 blocking therapies [110]. Finally, a combination of sh-circQSOX1 and anti-T-lymphocyte-associated antigen-4 (CTLA-4) could be more effective for overcoming the resistance to immune therapies mediated by Treg cells in CRC [111].

## CircRNA therapies

The use of RNA-based therapeutics may provide a potential treatment for various human diseases, including infectious diseases, cancers, and lipid-related diseases. For instance, mRNA vaccines can induce specific immune responses to protect against infectious diseases and cancers in animal models and humans [112]. Various RNA-based therapies, such as antisense oligonucleotides (ASOs), siRNAs, ASO anti-microRNAs (anti-miRs), miRNA mimics, miRNA sponges, circRNA therapies and CRISPR-Cas9-based gene editing, are performed and found to improve the quality of life and prolongs the lifespan of patients with various disease [113, 114]. The FDA and/or European Medicines Agency (EMA) have approved 11 RNA-based therapeutics targeting multiple patient tissues and organs [113]. Furthermore, their study and ours indicated that siRNA is a useful tool for silencing genes [115, 116]; four siRNA drug candidates (Patisiran, Givosiran, Lumasiran, and Inclisiran) have been approved by FDA and/or EMA [114]. Yu et al. designed synthesized chrysotile nanotubes (SCNTs) to encapsulate siRNA (SCNTs/si-circPRMT5) against the oncogenic circPRMT5 expression and then inhibited bladder cancer growth and metastasis [117]. Hence, SCNTs/si-circPRMT5 may have therapeutic value in treating bladder cancer.

Like other RNA therapeutics, circRNA has the potential effects of modulating gene expression or carrying out modular functions. CircRNAs served as miRNA sponges, further broadening the possibilities for inhibiting the oncogenic RNA function. For example, hsa_circ_001783 promoted breast cancer progression via sponging miR-200c-3p [118]. Synthetic circRNAs have attracted more attention due to their strong and stable translation in eukaryotic cells [119, 120]. Recently, Li et al. developed a novel circRNA vaccine platform to stimulate robust innate and adaptive immune responses for the anti-tumor effect in multiple mouse tumor models [121]. Qu et al. presented the circRNA vaccine against SARS-CoV-2 encoding the spike protein to protect against SARSCoV-2 infection [122]. Moreover, they also demonstrated the use of synthetic circRNAs to produce neutralizing antibodies against SARS-CoV-2 and hACE2 decoys to neutralize pseudovirus particles [122]. However, synthesized circRNAs still face many challenges for their development as therapeutic agents, such as avoiding sustained overexpression due to their exceptional properties, the production of highly purified artificial circRNAs, and their specific delivery. Therefore, further research should be required to address and overcome these challenges.

In another application, engineered circRNA purified by high-performance liquid chromatography demonstrated outstanding protein production quality in both quantity and stability production in eukaryotic cells [123]. Thus, circRNA is a viable alternative to linear mRNA. In addition to engineered circRNAs, CircaRNA-based aptamers are produced by twister-optimized RNA for durable overexpression (Tornado) that circularizes RNA to produce aptamers capable of binding proteins [124]. Despite significant progress in the research and application of circRNAs, most candidates are currently in the discovery or preclinical stages. So far, no circRNA therapeutic candidate has entered clinical trials. Numerous medical and research applications exist for CircRNA, including cancer therapy, protein replacement therapy, and prophylactic vaccines. In addition, it is important to note that circRNAs can also be targeted for modulation using other methods, such as CRISPR-Cas9 or siRNA, and native circRNAs can be used as biomarkers or sponging agents for various diseases, especially cancer. Nevertheless, RNA therapeutics, like mRNA-based therapies, can be translatable to circRNA-based therapies and provide valuable insights into the development of circRNAs as therapeutic agents.

## Challenges and future perspectives

Although the biological role of circRNA in CRC has received increased research attention recently, the role of circRNAs in CRC treatment has not yet been extensively explored, indicating the need for further studies. For instance, the biological and molecular mechanisms of only a few circRNAs in CRC have been elucidated. Further research is needed to determine the exact mechanisms of circRNA circularization, degradation, extracellular transport, and subcellular localization in CRC. Owing to the potential for alternative splicing of pre-mRNA [125], the internal structure of circRNAs remains unclear. Recently, a novel algorithm called CircSplice has been developed and is capable of identifying alternate splicing in circRNAs and comparing distinct circRNA splicing events [126]. The patterns of cancer-specific circRNA alternative splicing (circ-AS) could be characterized using CircSplice, providing a promising resource for elucidating the regulation and functional implications of circRNAs in cancers. Several studies on circRNAs mainly focus on their role as miRNA sponges, RBP sponges, and regulators of mRNA expression. However, circRNA function may also be regulated by mechanisms other than those mentioned above. Additionally, studies are yet to clarify whether circRNAs can be simultaneously regulated by different molecular mechanisms.

Furthermore, several studies have shown that TME is closely related to colorectal tumor initiation and progression [127]. In several cancers, including CRC, the characteristics of the TME strongly influence tumor invasion, metastasis, proliferation, and drug resistance [128]. Therefore, research on the role of circRNAs in TME in patients with colorectal cancer may provide potential novel biomarkers and therapeutic targets for CRC treatment. Currently, the clinical applications of circRNA biomarkers are limited because of their lack of sensitivity and specificity, indicating the need for further studies using standardized techniques and bioinformatics approaches.

Although some circRNAs are located in the nucleus (such as intronic and exon-intron circRNAs) [40], most circRNAs accumulate in the cytoplasm [23]. Numerous studies have demonstrated that circRNAs can be exported from the nucleus to the cytoplasm to perform regulatory functions [129, 130]. However, it is unclear how circRNAs are exported from the nucleus since they lack characteristics used by RNA export pathways. Surprisingly, Huang et al. discovered a novel, evolutionarily conserved pathway for circRNA export [131]. Deleting the Drosophila DExH/D-box helicase Hel25E accumulates long (more than 800 nucleotides) circular RNAs in the nucleus [131]. In recent studies, DDX39B and DDX39A, components of TREX, Exportin 4 (XPO4), are shown to regulate the export of exonic circular RNAs, while NXF1/NXT1 determines the export of cytoplasmic circular RNAs [132]. The NXF1-NXT1 pathway modulates toxic DPR production through the nuclear export of circular introns [129]. Researchers have identified DDX39A and DDX39B as regulators of ecircRNA nuclear export [131]. Recent studies have shown that DDX39B could unwind R-loops, and DDX39 participated in ecircRNA export by resolving ciR-loops [133]. In addition, Chen et al. reported that the conserved XPO4 is linked to the nuclear export of circRNAs in metazoans [134]. Knockout of XPO4 led to the ecircRNA accumulation in the nucleus [134]. Moreover, Chen et al. identified an N^6^-methyladenosine (m^6^A) modified circRNA, which can affect cytoplasmic export and CRC development. For example, m^6^A modification of circNSUN2 increased its export to the cytoplasm, forming a cirNSUN2/IGF2BP2/ HMGA2 RNA-protein ternary complex in the cytoplasm and promoting CRC by enhancing the stability of HMGA2 mRNA [69]. Thus, export of circRNA from the nucleus to the cytoplasm is required for its proper function.

Finally, further studies are required to develop effective circRNA delivery systems to tumor cells to regulate cancer progression without immune rejection and with sustained long-term effects. The applications of circRNAs may be remarkably improved by exosomes. Several eukaryotic cells and cancerous cells release extracellular vesicles, known as exosomes, that mediate intercellular communication via the transport of signaling molecules, including circRNAs, to cancer cells [135]. Exosomes have been confirmed to contain circRNAs, including circPACRGL, circSHKBP1, circUHRF1, and ciRS-122 [136]. Interestingly, serum exosomal circSATB2 levels can be used to identify patients with lung cancer with high sensitivity and specificity [78]; however, the biological functions of exosomal circRNAs in CRC requires further study.

## Conclusions

This review extensively discusses the biogenesis, characteristics, and mechanisms of circRNA in CRC. Particularly, the potential clinical applications of circRNA as biomarkers for CRC diagnosis and prognosis and as therapeutic targets for CRC treatment were highlighted. However, the understanding of the activities of circRNAs and how they initiate CRC is lacking, indicating the need for further research.

## Data Availability

There are no experimental datasets, given that this is a review article that is prepared based on a literature review.
